# Reducing protein regulator of cytokinesis 1 as a prospective therapy for hepatocellular carcinoma

**DOI:** 10.1038/s41419-018-0555-4

**Published:** 2018-05-10

**Authors:** Xinran Liu, Yangkai Li, Lijing Meng, Xin-Yuan Liu, Anlin Peng, Yuchen Chen, Chengyu Liu, Hong Chen, Sheng Sun, Xiaoping Miao, Yu Zhang, Ling Zheng, Kun Huang

**Affiliations:** 1 Tongji School of Pharmacy, Huazhong University of Science & Technology, Wuhan, 430030 China; 2 Centre for Biomedicine Research, Wuhan Institute of Biotechnology, Wuhan, 430074 China; 3 Tongji Hospital, Tongji Medical College, Huazhong University of Science & Technology, Wuhan, 430030 China; 4 Institute of Biochemistry and Cell Biology, Shanghai Institutes for Biological Sciences, Chinese Academy of Sciences, Shanghai, 200031 China; 5 The Third Hospital of Wuhan, Wuhan, 430060 China; 6 Tongji School of Public Health, Huazhong University of Science & Technology, Wuhan, 430030 China; 7 Hubei Key Laboratory of Cell Homeostasis, College of Life Sciences, Wuhan University, Wuhan, 430072 China

## Abstract

Proteins that bind to microtubule are important for cell cycle, and some of these proteins show oncogenic characteristics with mechanisms not fully understood. Herein we demonstrate overexpression of protein regulator of cytokinesis 1 (PRC1), a microtubule-associated regulator of mitosis, in human hepatocellular carcinoma (HCC). Moreover, upregulated PRC1 is associated with lower survival rates of HCC patients. Mechanistically, reducing PRC1 blocks mitotic exit of HCC cells at telophase in a spindle assembly checkpoint independent manner, and acts synergistically with microtubule-associated agents (MTAs) to suppress p53-wt or p53-null HCC cells in a p53- or p14ARF-dependent manner; while overexpressing PRC1 increases the resistance of HCC to taxol. A combined treatment of taxol/shPRC1 results in 90% suppression of tumor growth in subcutaneous HCC xenograft models. In orthotopic xenograft mice, reducing PRC1 significantly alleviates HCC development and hepatic injury. Together, our results suggest a dual-mitotic suppression approach against HCC by combining MTAs with cytokinesis inhibition, which blocks mitosis at both metaphase and telophase.

## Introduction

Hepatocellular carcinoma (HCC) results in approximately 600,000 deaths worldwide annually^1^. Microtubule-targeting agents (MTAs), such as taxanes and epothilones, bind with microtubules, altering their dynamics, triggering the spindle assembly checkpoint (SAC) and preventing cells from entering anaphase, which causes mitotic arrest^2^. However, because of drug resistance or insensitivity, applications of these chemotherapeutic agents are limited for HCC treatment^3^.

During MTAs induced mitotic arrest, cancer cells either die or exit mitosis by slipping into G1 phase with mis-segregated chromosomes, displaying increased tumorigenicity and resistance to MTAs^4–6^. Therefore, targeting mitotic exit represents an important therapeutic approach to overcome MTA insensitivity^7, 8^. Presently, most strategies block mitotic exit at the metaphase-to-anaphase transition via SAC^9^; unfortunately, reduced SAC activity in cancer cells is commonly found^10^, new approaches against MTA-resistant cancers are needed.

Protein regulator of cytokinesis 1 (PRC1) is a microtubule binding protein required for the completion of cytokinesis at telophase^11^. PRC1 was previously identified as a substrate of cyclin-dependent kinase (CDK) and a regulator of mitotic spindle midzone formation^12, 13^. In addition to its essential functions in mitosis, upregulated PRC1 has been observed in bladder and breast cancers^11, 14^. Recently, it has been reported that PRC1 promotes early recurrence of HCC in association with the Wnt/beta-catenin signaling^15^. However, whether PRC1 affects cancer development through its role in mitosis is unclear.

Here we demonstrated that PRC1 knockdown inhibits HCC proliferation through blocking cytokinesis in an SAC-independent manner, which enhances the toxicity of multiple MTAs to HCC with synergistic effect. Based on these findings, we tested a dual-mitotic suppression strategy against HCC by combining MTAs with blocking cytokinesis.

## Results

### PRC1 is overexpressed in HCC

**Fig. 1:**
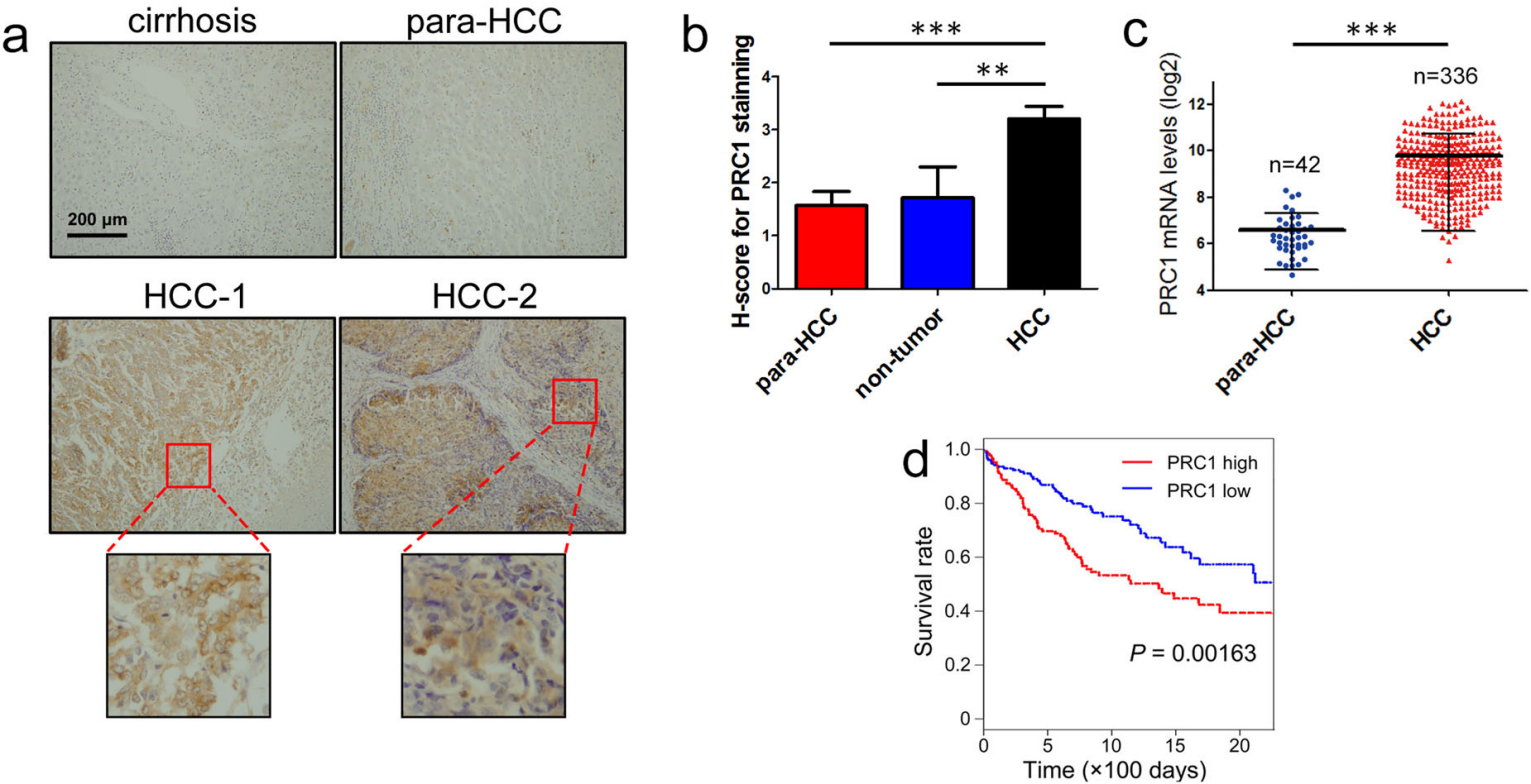
Overexpression of PRC1 in HCC samples. **a** Representative pictures of PRC1 immunohistochemical staining on clinical samples. **b** The H-score of PRC1 for different groups; presented as mean ± SD. **c** The mRNA levels of PRC1 in HCC and para-HCC tissues (data from TCGA). **d** The survival rates of HCC patients with high PRC1 expression levels (red) and low levels (blue) (the cutoff for determining high vs low levels of PRC1 is the midpoint, data from TCGA). (**P < 0.01, ***P < 0.001)

Immunohistochemistry assays of 100 clinical samples were analyzed. Based on the range and intensity of Histo-score (H-score) of PRC1 staining, overexpressed PRC1 in both nuclei and cytoplasm was observed in HCC tissues compared to para-HCC tissues and non-malignant tissues (Table S2 and Fig. 1a, b), which is consistent with a previous report^15^. We further analyzed data of 336 HCC and 42 non-tumor samples of patients from The Cancer Genome Atlas (TCGA), a significant upregulation of *PRC1* in HCC tissues was suggested (Fig. 1c). Importantly, the overall 5-year survival rates of HCC patients with *PRC1* levels above average were significantly lower than those with lower levels of *PRC1* (*P* = 0.00163, Fig. 1d), suggesting a correlation between PRC1 upregulation and poor prognosis.

### Reduction of PRC1 inhibits proliferation of HCC cells

**Fig. 2:**
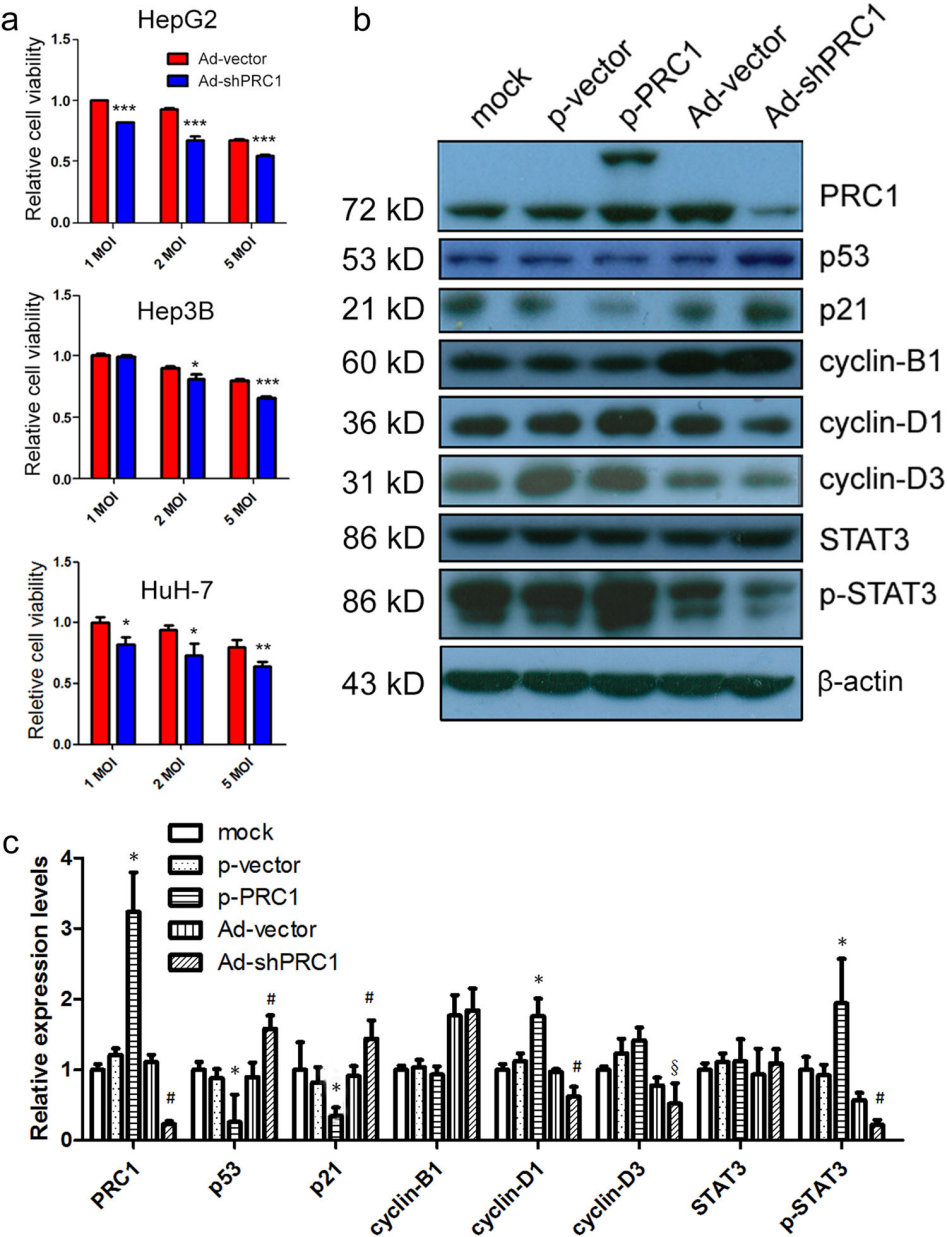
Inhibitory effect on cell growth by reduction of PRC1 in HCC cells. **a** Relative cell viability of HepG2 (top), Hep3B (middle), and HuH-7 (bottom) cells after infection with Ads of indicated MOIs using MTT assays. Value of cells receiving Ad-vector at MOI = 1 was arbitrarily set at 1. *n* = 3 independent experiments. **b** Western blots for indicated proteins in HepG2 cells transfected with p-PRC1 or p-vector and in HepG2 cells infected with Ad-shPRC1 or Ad-vector. **c** Densitometric analysis of western results. *P < 0.05 compared to p-vector treated group; ^#^*P* < 0.05 compared to Ad-vector-treated group, ^§^0.1 < *P* < 0.15 compared to Ad-vector-treated group

We next reduced the expression of endogenous PRC1 in HCC cells by using a cancer-specific adenoviral system (Fig. S1a)^16^. To evaluate the efficiency of Ad-shPRC1, HepG2 and Hep3B cells were infected. Forty-eight hours after infection, both the mRNA and protein levels of *PRC1* were significantly reduced in HepG2, Hep3B, and HuH-7 cells (*P* < 0.001, Fig. S1b, c), and a concentration-dependent knockdown efficiency by Ad-shPRC1 was observed (Fig. S1b). Moreover, MTT assay results demonstrated that knockdown of PRC1 by adenoviral system significantly inhibited the viability of HepG2, Hep3B, and HuH-7 cells (Fig. 2a).

To determine the effects of PRC1 level on mitotic arrest-associated molecules, HepG2 were infected with Ad-shPRC1 or Ad-vector, or transfected with empty plasmid or plasmid carrying PRC1. Western blots confirmed the expression of fusion protein (exogenous PRC1 tagged with EGFP, band on top of the endogenous PRC1), as well as reduction of endogenous PRC1 by Ad-shPRC1 (Fig. 2b). Knockdown of PRC1 enhanced the expression of p53, a tumor suppressor that controls cell cycle; and upregulated p21, a cell cycle inhibitor downstream of p53 that controls the G1/S transition (Fig. 2b, c)^17^. Consistently, overexpression of exogenous PRC1 significantly reduced such upregulations (Fig. 2b, c). Meanwhile, cyclin D1 and D3, two G1 phase cyclins that promote cell cycle progression, were decreased by PRC1 knockdown, and vice versa (Fig. 2b, c). In addition, we observed the levels of cyclin D1 and phosphorylated STAT3 were both reduced following reduction of PRC1, while overexpression of PRC1 showed opposite effects (Fig. 2b, c). Notably, altered PRC1 expression did not affect cyclin B1 (Fig. 2b, c), which is an M-phase cyclin regulating the metaphase/anaphase transition, suggesting that shPRC1 possibly induces a non-metaphase mitotic arrest. Recent study suggests that in p53-null or p53-mutated cancer cells, p14ARF may partly compensate the tumor suppressor role of p53^18, 19^. Consistently, we observed that p14ARF and cell cycle inhibitor p21 were both upregulated by shPRC1 in Hep3B (p53-null) and HuH-7 (p53-loss-of-function-mutation) cells by Ad-shPRC1 (Figs. S2a, b).

### Reducing PRC1 sensitizes HCC cells to taxol by blocking telophase

**Fig. 3:**
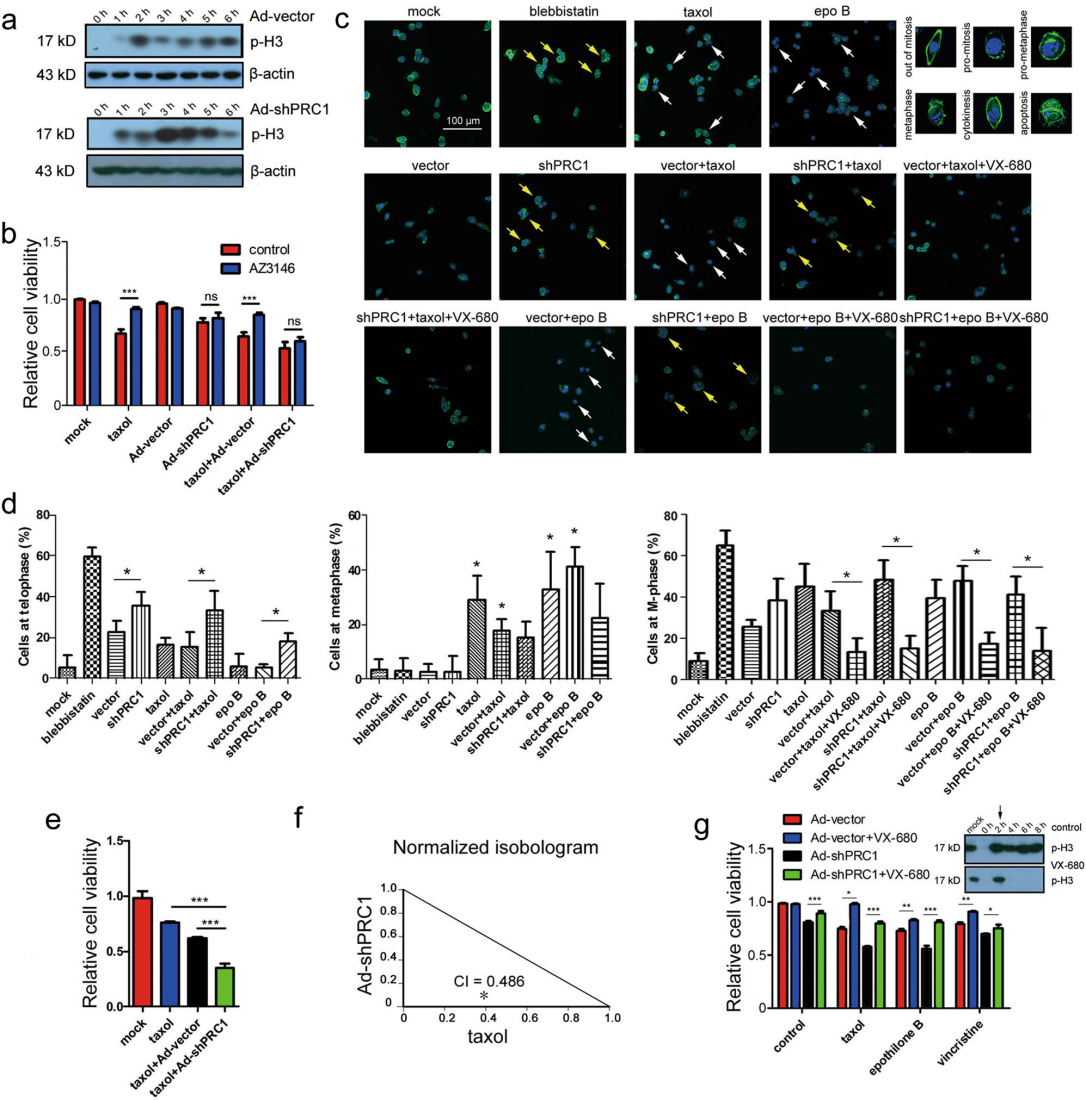
Ad-shPRC1 enhances taxol toxicity to HCC cells by blocking mitotic exit at telophase. **a** The temporal variation in phosphorylated H3 after synchronization by VX-680 measured using western blots. **b** MTT results of indicated treatments. *n* = 3 independent experiments. **c** Representative confocal microscopy images of cells at different phases of mitosis following different treatments. Typical cell morphologies of different mitotic phases are presented at corner of top right. Blue, DAPI-stained nuclei; green, phalloidin, stained microtubules; yellow arrows, cells at cytokinesis; white arrows, cells at pro-metaphase. Magnification, ×100. **d** Percentage of cells arrested at telophase, pro-metaphase or M-phase under different treatments (epo, epothilone B). Results respond to three slides of each group. **e** Cell viability of HepG2 cells with indicated treatments using MTT assays. Value of mock-treated cells was arbitrarily set at 1. *n* = 3 independent experiments. **f** The combination index of Ad-shPRC1/taxol on HepG2 cells. **g** Effect of VX-680 treatment on cell growth measured by MTT assays. Value of cells receiving Ad-vector was arbitrarily set at 1. *n* = 3 independent experiments. Phosphorylated H3 level shown at the top-right corner. (*P < 0.05, **P < 0.01, ***P < 0.001)

PRC1 is a critical protein associated with cytokinesis, and its reduction cause defects in cytokinesis^11, 12^. Consequently, we determined whether shPRC1 blocks the cell cycle in HCC cells. HepG2 cells were synchronized at G2/M or M-phase, respectively, by VX-680 or nocodazole treatment (Fig. S3a). We measured the length of mitosis by checking the phosphorylation level of histone H3 (p-H3), an M-phase indicator^20^. In VX-680-induced synchronization, significantly delayed mitotic exit of cells receiving shPRC1 was observed as demonstrated by the dropped p-H3 levels (6 vs 3 h, Fig. 3a). When cells were released from the nocodazole-induced synchronization, the p-H3 level in shPRC1-treated cells dramatically went down at 4–6 h after release, much longer than the controls (within 2 h, Fig. S3b). Together, these results suggest a prolonged mitotic arrest induced by PRC1 knockdown.

SAC activation is an essential mechanism for certain MTA treatments; however, its surveillance can be evaded by some cancer cells^2, 10^. To explore whether SAC activation is required for shPRC1-induced growth inhibition of HCC cells, an SAC inhibitor AZ3146 was used^21^. AZ3146 showed little effect on cell viability per se, but significantly attenuated the toxicity of taxol (one of commonly used MTAs) to HepG2 cells (Fig. S3c), which was consistent with a previous report that MTAs-induced growth inhibition requires SAC activation^22^. However, the viability of cells receiving Ad-shPRC1 or Ad-shPRC1/taxol combination was unaffected by AZ3146 treatment (Fig. 3b), indicating that the suppressive function of shPRC1 is independent of SAC activation.

To demonstrate the morphology of HCC cells after PRC1 knockdown, a time-lapse microscopy analysis was performed. In shPRC1-treated HepG2 cells, multinuclear were induced and formation of apoptotic bodies followed shortly, indicating cytokinesis failure and subsequently apoptosis in these cells (Fig. S3d). Next, confocal microscopy was applied to examine the cell phase that shPRC1 suppresses. Cells rapidly entered M-phase 30 min after being released from G2/M synchronization by VX-680 (*P* < 0.05, Fig. S3e), indicating a successful synchronization. At 8 h after releasing from synchronization, shPRC1 induced a significant increase in the number of cells arrested at telophase (shown as cytokinesis, yellow arrows, Fig. 3c, d), which were similar to the cells receiving blebbistatin, an agent that induces mitotic defects of cytokinesis (Fig. 3c, d)^23^. On the other hand, taxol and epothilone B, the latter is an MTA in clinical trials for breast and ovarian cancers^24^, mainly caused arrest at pro-metaphase/metaphase (shown as pro-metaphase, white arrows, Fig. 3c, d). Combined treatment of Ad-shPRC1 with taxol/epothilone B significantly increased the number of cells with defects in cytokinesis compared with that of taxol or epothilone B alone (*P* < 0.05, Fig. 3c, d). Additionally, taxol, epothilone B, Ad-shPRC1 or their combinations rapidly reduced the number of cells in M-phase by VX-680 re-treatment, which induces slippage of the mitotic arrested cells as previously reported (*P* < 0.05, Fig. 3d)^25^. Together, these results demonstrate that reducing PRC1 blocks mitotic exit at telophase, while taxol/epothilone B mainly suppress mitosis at metaphase.

Some cancer cells, such as HCC, can exit mitotic arrest by prematurely entering anaphase to survive anti-mitotic therapy^26^. Consequently, we propose that a dual-mitotic suppression approach, which blocks mitosis at both metaphase and telophase using a taxol/Ad-shPRC1 combination, should bring synergistic effects against HCC. To test this hypothesis, the efficacy of combined taxol/Ad-shPRC1 treatment was investigated. MTT assays indicated that the taxol/Ad-shPRC1 combination resulted in significantly enhanced cytotoxicity compared with taxol or Ad-vector alone (Fig. 3e); while overexpression of PRC1 significantly protected the HepG2 cells from taxol (*P* < 0.001, Fig. S3f), suggesting that PRC1 plays a critical role in the toxicity of taxol to HCC cells. Moreover, the combination index (CI) suggests the combination of shPRC1/taxol brings synergistic suppressive effects on the viability of HepG2 cells (CI = 0.486, Fig. 3f).

To further assess whether shPRC1 enhances the cytotoxicity of other mitotic drugs by blocking mitotic exit, HepG2 cells were synchronized at G2/M-phase by VX-680 and treated with taxol, epothilone B or vincristine. shPRC1 significantly increased the toxicity of all three MTAs to HCC cells (Fig. 3g); while after mitotic exit being restored by VX-680 re-administration, combined treatments reversed the decreased cell viability (*P* < 0.001 and *P* < 0.05, Fig. 3g). Moreover, the CI analysis also suggested a synergistic suppressive effect of the combinational treatment of shPRC1 with epothilone B or vincristine on HepG2 cells (CI = 0.796 for shPRC1/epothilone B and CI = 0.650 for shPRC1/vincristine, Fig. S3g). Together, these data demonstrate that the induced MTA sensitivity of HCC cells by shPRC1 depends on blocking mitotic exit.

Since STAT3 is an oncogene reported to play a critical role in cell cycle and proliferation regulation^27^, we studied whether STAT3 activation regulates the shPRC1-induced cytokinesis block and taxol sensitivity of HCC cells. The results demonstrated that neither activation (by interlukin-6 or epidermal growth factor^28^) nor inhibition (by cyt387 or cryptotanshinone^29, 30^) of STAT3 phosphorylation affected shPRC1-induced cytokinesis defect or taxol sensitivity of HepG2 cells (Fig. S4a–c), which suggest that such PRC1-mediated biological functions are independent of STAT3 phosphorylation.

### Reduction of PRC1 sensitizes HCC cells to taxol through the p53 pathway

**Fig. 4:**
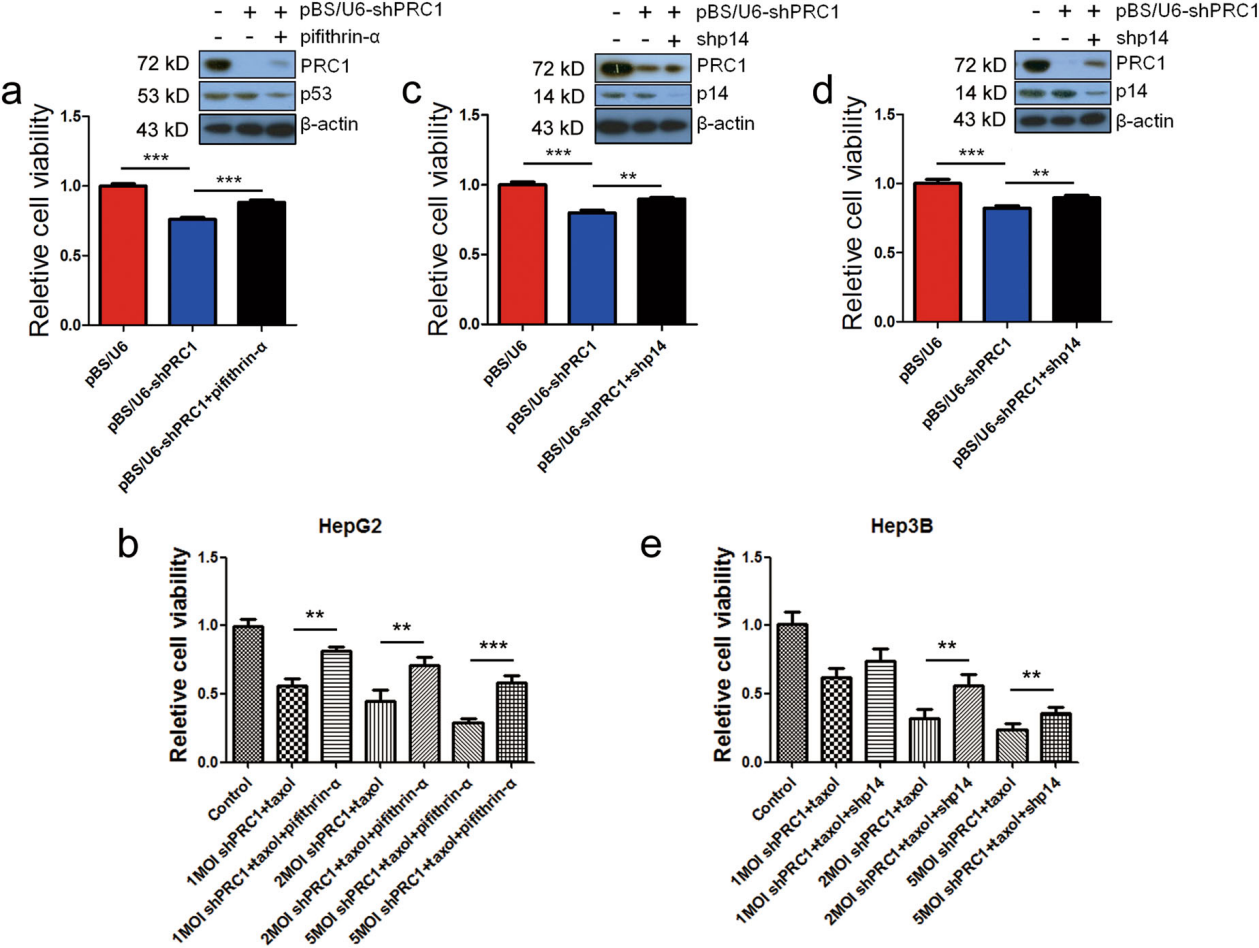
Reducing PRC1 attenuates the proliferation of HCC cells in a p53/p14-dependent manner. **a** Cell viability and the western blots for p53 and PRC1 (right-top corner) of HepG2 cells with indicated treatments. **b** MTT results of HepG2 cells with indicated treatments. Taxol concentration, 10 μM. Value of control cells was arbitrarily set at 1. **c**, **d** Cell viability and the western blots for p14ARF and PRC1 (right-top corner) of Hep3B (**c**) and HuH-7 **(d**) cells with indicated treatments. **e** MTT results of Hep3B cells with indicated treatments. Taxol concentration, 10 μM. Value of control cells was arbitrarily set at 1 (*P < 0.05,**P < 0.01, ***P < 0.001)

The tumor suppressor p53 plays a critical role in the development of drug resistance in cancer cells^31^, and its accumulation during mitotic arrest triggers apoptosis following mitotic slippage^32^. To explore whether the reduced cell viability by shPRC1 is dependent on the p53 pathway in p53-wt cells, a p53-specific inhibitor pifithrin-α was used^33^. In addition to the Ad-vector, PRC1 reduction was also achieved by shPRC1 plasmid transfection to test the growth inhibition effects of shPRC1. The results showed that shPRC1 plasmid transfection also suppressed the viability of HepG2 cells (p53-wt) (Fig. 4a), while pifithrin-α attenuated the viability suppression of HepG2 cells by shPRC1 plasmid (Fig. 4a). Notably, the toxicity of the taxol/Ad-shPRC1 combination on HepG2 cells was also markedly reduced by pifithrin-α (Fig. 4b), suggesting shPRC1 suppresses the p53-wt HCC cells in a p53-dependent manner.

Similar to our findings using Ad-shPRC1, PRC1 reduction by shPRC1 plasmid transfection also induced cell growth inhibition of Hep3B and HuH-7 cell lines (Fig. 4c, d). Since p14ARF may partially substitute for p53 in inhibiting the proliferation of p53-null and p53-mutant cancer cells as described above, we hypothesized that reduction of cell viability by shPRC1 in p53-null/mutant cells may depend on p14ARF. As expected, we observed a decreased cytotoxicity of shPRC1 plasmid to Hep3B and HuH-7 cells in double knockdown cells (p14-shRNA + shPRC1 plasmids) compared to shPRC1 plasmid per se (Fig. 4c, d). In addition, reduction of p14ARF significantly restored the viability of Hep3B cells receiving Ad-shPRC1/taxol combined treatment (*P* < 0.001, Fig. 4e), suggesting that p14ARF plays a critical role in the tumor suppressor role of shPRC1 in p53-null /mutant HCC cells.

To further study the role of p53/p14ARF in shPRC1-induced mitotic arrest, we reduced PRC1 in HepG2 and Hep3B cells respectively and restored mitotic exit by VX-680 re-administration. The results showed that shPRC1-induced mitotic arrest led to upregulation of p53/p14ARF; however, when the cells were released from mitotic arrest, the levels of p53/p14ARF significantly reduced even in the presence of shPRC1 (Fig. S4d). Similarly, foreign PRC1, which boosted the proliferation process, downregulated p53/p14ARF, whereas subsequent treatment of the mitotic inhibitor Nocodazole significantly re-enhanced p53/p14ARF (Fig. S4e), suggesting that PRC1 affects p53/p14ARF through regulating the mitotic process.

### Reducing PRC1 inhibits the growth of HCC xenografts

**Fig. 5:**
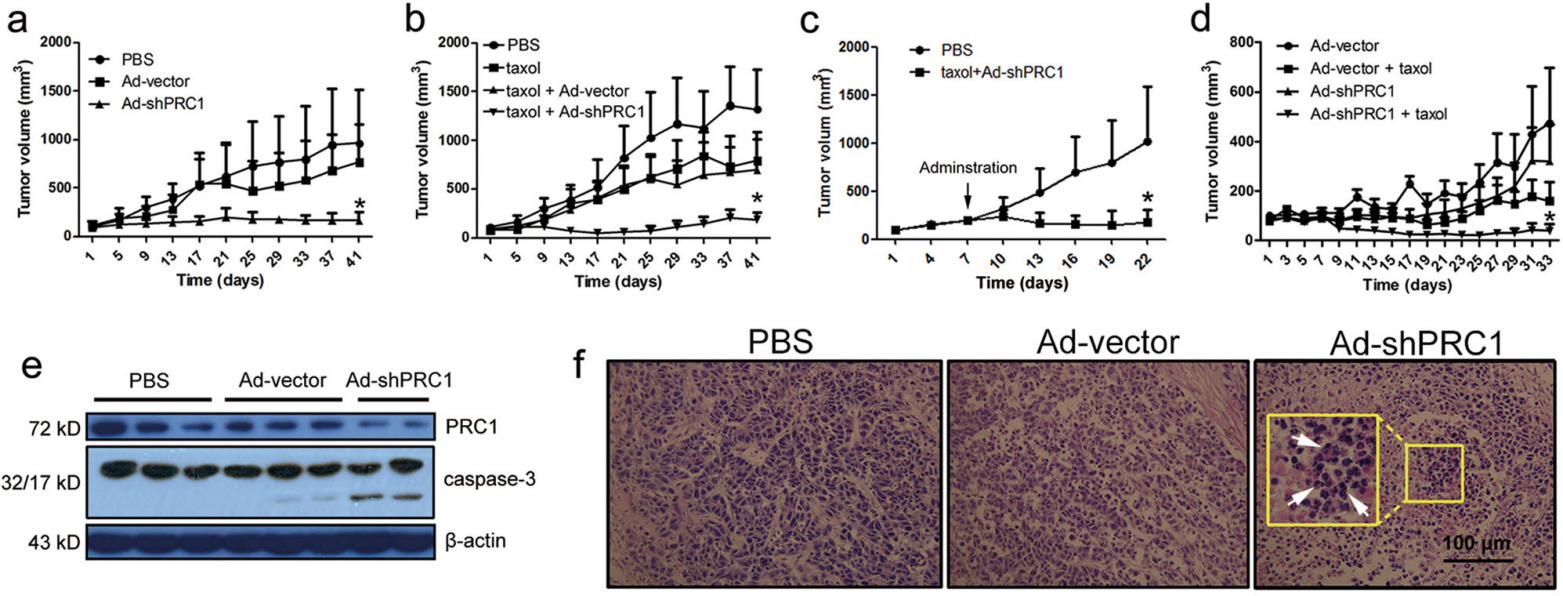
Reducing PRC1 suppresses growth of HCC xenografts and improves efficacy of taxol against HCC. **a**–**d** Tumor volumes of the HCC xenografts of HepG2 (**a**−**c**), or HuH-7 (**d**) cells receiving different Ads and/or taxol. The mice started to receive injections when the tumors reached 80 mm^3^ (**a**, **b**, **d**) or 200 mm^3^ (**c**) in volume. **a**−**c** are independent experiments. **e** Western blots for PRC1 and caspase-3 of different HCC xenografts. **f** H&E staining of xenograft samples; white arrows indicate the apoptotic cells with nuclear fragmentation. (*P < 0.05)

To test the dual-mitotic suppression strategy in vivo, subcutaneous xenograft models were used, and when the tumors reached 80 mm^3^ in volume, mice were randomly divided and started to receive injection. At the end point of the HepG2 xenograft experiments, the mean tumor volumes of the PBS and control Ad-vector groups were 963 ± 548 mm^3^ and 767 ± 392 mm^3^, respectively; in comparison, the mean tumor volume of Ad-shPRC1 group was 169 ± 83 mm^3^ (*P* < 0.05, Fig. 5a). In another batch of xenograft experiment, the efficacy of taxol mono-treatment was relatively low, while the mean tumor volume of the taxol/Ad-shPRC1 group was 51 ± 63 mm^3^, demonstrating a higher efficacy compared to taxol or Ad-shPRC1 alone (*P* < 0.05, Fig. 5b). To further test the potent efficacy of the dual therapy, the subcutaneous HepG2 xenografts started to receive taxol/Ad-shPRC1 when they reached 200 mm^3^ on average, and significant tumor regression was observed (Fig. 5c). Furthermore, to study the in vivo tumor suppressor role of shPRC1 on HCC with p53-mutant, the mouse model of HCC xenograft by HuH-7 cells was used, and the dual therapy of taxol/Ad-shPRC1 showed the best efficacy, suggesting shPRC1 increases taxol sensitivity of p53-mutant HCC cells (Fig. 5d).

Moreover, an obvious reduction of PRC1 and cleaved caspase-3 was induced by Ad-shPRC1 injection, suggesting that PRC1 knockdown results in apoptosis of HCC cells in vivo (Fig. 5e). H&E staining also revealed large areas of apoptotic cells with visible nuclear fragmentation and chromatin condensation in tumors injected with Ad-shPRC1 (arrows, Fig. 5f). Together, these results suggested that reducing PRC1 has a strong anti-cancer effect against both p53-wt and p53-mutant HCCs in vivo, especially when combined with taxol.

### PRC1 knockdown inhibits orthotopic HCC xenograft development and restores hepatic function

**Fig. 6:**
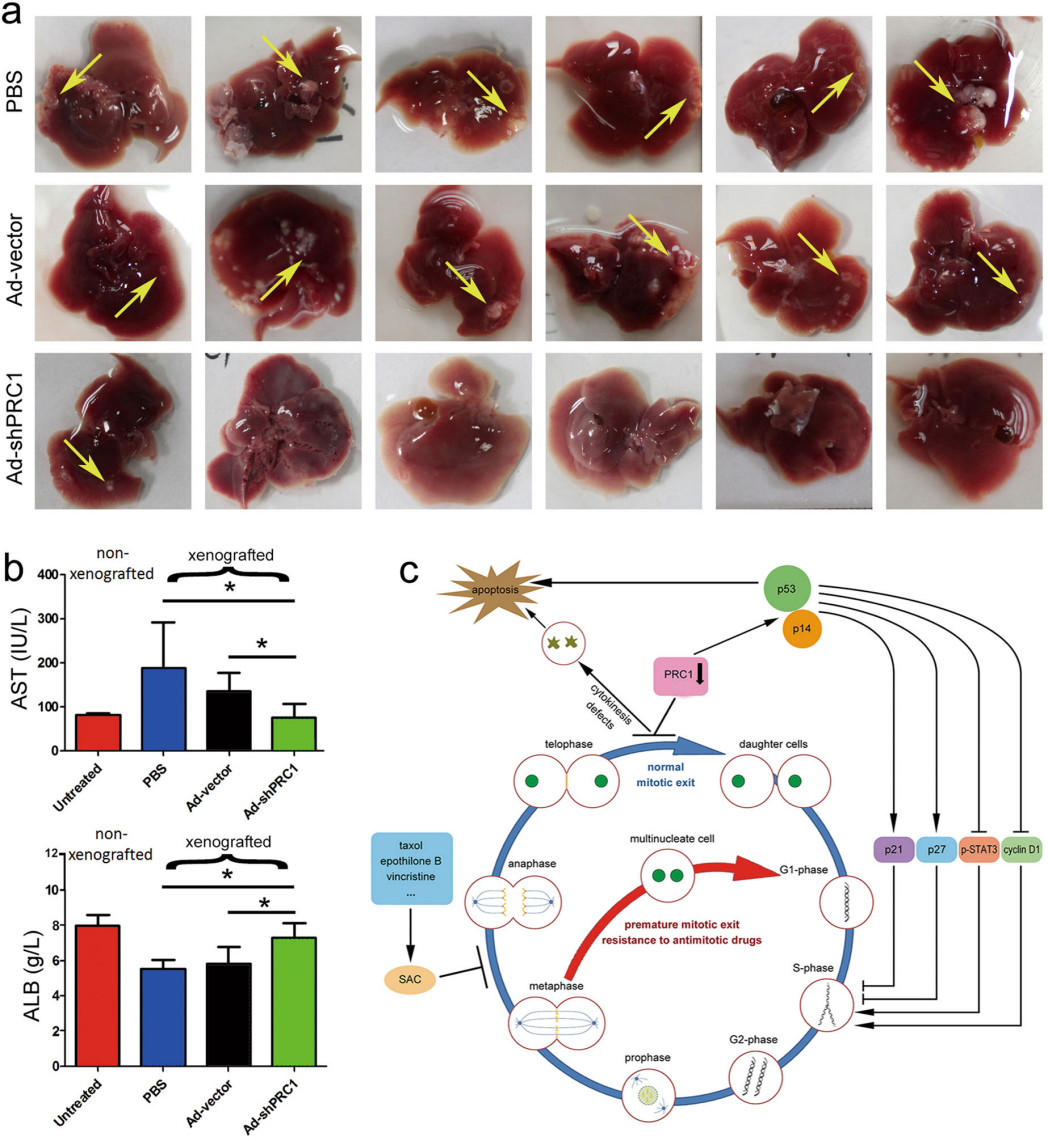
Ad-shPRC1 treatment suppresses development of orthotopic HCC xenografts and protects hepatic function. **a** The livers with orthotopic HCC xenografts under different treatments; tumor nodules are indicated with yellow arrows. **b** Serum AST and ALB levels of the orthotopic HCC xenografted mice at 21 days after receiving different treatments. **c** A potential mechanism for dual-mitotic suppression strategy for treating HCC. (*P < 0.05)

An orthotopic HCC xenograft model was further established to study the anti-HCC effect of Ad-shPRC1 in situ. Four weeks after engraftment, all mice (6 of 6) in the control groups receiving PBS or Ad-vector developed obvious tumor nodules in the liver (arrows, Fig. 6a), and four mice from each group developed ascites (data not shown). In contrast, only one of six livers from the Ad-shPRC1 group showed visible tumor nodules (Fig. 6a), and only one mouse receiving Ad-shPRC1 treatment developed ascites. To further evaluate whether reducing PRC1 protects hepatic function, blood samples were collected and analyzed. Eighteen days after Ad-shPRC1 injections, the abnormal aspartate transaminase (AST) and albumin (ALB) levels caused by the HCC xenograft were restored (Fig. 6b).

## Discussion

SAC activation is critical for MTAs to suppress mitosis, whereas drug-resistant cancer cells can exit mitosis by prematurely entering anaphase to evade SAC (Fig. 6c). Reducing PRC1, which blocks cytokinesis at telophase, may target the cells escaping from the metaphase arrest bringing synergistic antitumor effects to MTAs treatment (Fig. 6c). With this rationale, we developed a dual-mitotic suppression approach that blocks mitotic exit at both metaphase and telophase using a combination of taxol and shPRC1. Our results suggest that this strategy provides excellent antitumor effects against HCC both in vitro and in vivo.

Evidence suggests that proteins involved in spindle assembly are connected with the sensitivity of cancer cells to MTAs^34^. For example, upregulation of KIFC3 and MCAK, which are microtubule-binding molecular motors, represents MTA resistance of breast cancer^3, 35^. Moreover, in addition to microtubule-binding proteins that affect SAC, some cytokinesis-associated proteins or agents may also enhance the efficacy of MTAs. For instance, mulberry water extract acts synergistically with taxol triggering cytokinesis defects and subsequently apoptosis in bladder cancer cell^36^. However, whether cytokinesis is responsible for the enhanced cytotoxicity is unknown. Here we demonstrate that reducing PRC1 enhances HCC sensitivity to MTAs through blocking cytokinesis. Our work raises the possibility that in addition to the known spindle-associated proteins, proteins essential for cytokinesis may also serve as novel antitumor targets. It will be interesting to study whether these agents are also potential candidates for treating MTA-resistant cancers.

In addition to cytokinesis and SAC, a number of mitotic components that function at different cell cycle phases have been suggested as targets for cancer therapy. For example, a therapy targeting CDK1/2, which has entered clinical trials to treat colon cancer and acute leukemia, abrogates G2/M transition and induces G2 suppression^37, 38^; tetraploidy checkpoint and hippo tumor suppressor pathway determine the fate of mitotic slipped cells at G1 phase^4, 5, 39, 40^. Since these regulators function in a broad range of cancers, we propose that simultaneously triggering multiple checkpoints of different mitotic phases may achieve good outcomes. Here a combinatorial HCC therapy was tested as a proof of concept, it will be interesting to test other resistant cancers, such as breast and colon cancers^3, 35, 37^, with this strategy.

## Materials and methods

### Cell lines and tissue specimens

Human hepatocellular carcinoma cell lines Hep3B, HepG2, HuH-7 were obtained from the Cell Bank of Type Culture Collection of the Chinese Academy of Sciences (Shanghai, China) or China Center for Type Culture Collection (Wuhan, China). HEK293 (human embryonic kidney cell line) was obtained from Microbix Biosystems Inc. (Toronto, Ontario, Canada). Cell lines were grown in Dulbecco’s modified Eagle’s medium (GIBCO BRL, Grand Island, NY) supplemented with 10% FBS as previously described^41^. Human samples, including 14 non-malignant liver diseases (cirrhosis, choledocholithiasis, hepatapostema, cholangitis, and cavernous hemangioma) and 50 HCC and 36 adjacent tissues were obtained from Union Hospital, Tongji Medical College of Huazhong University of Science and Technology (Wuhan, China). The protocol for acquisition of tissue specimens was approved by the Institutional Review Board of Union Hospital; human sample collection procedures were in accordance with the established guidelines.

### Information for plasmids used in the present study

To produce shRNAs in mammalian cells, we used a previously developed system^16^. Based on the shRNA targeting sequence in the coding region of the human PRC1 gene, two single-strand oligonucleotides were designed and coded as follows:

*shPRC1* Forward: 5′-GCATATCCGTCTGTCAGAAGAAGACTTCTGACAGACGGATATGCCTTTTTG-3′

*shPRC1* Reverse: 5′-AATTCAAAAAGGCATATCCGTCTGTCAGAAGTCTTCTTCTGACAGACGGATATGC-3′

These single-strand oligonucleotides were annealed to generate the double-stranded oligonucleotides that were cloned into the *Apa*I (blunted) and *Eco*RI sites of pBS/U6, to generate the plasmid pBS/U6-shPRC1 (shPRC1 plasmid).

The plasmid for the fusion PRC1, which is tagged with EGFP with a molecular weight of 100 kD, was obtained from GeneChem Co. (Shanghai, China). shp14 plasmid is a gift from Prof. X. Liu (Shanghai Institute for Biological Sciences, Chinese Academy of Sciences).

### Generation, purification, and titration of the oncolytic adenovirus (Ad)

Generation of the oncolytic Ads was performed as previously reported^16, 42^. Briefly, to generate Ad-shPRC1, pBS/U6-shPRC1 was digested with *Bam*HI, and the U6-shPRC1 expression cassette was inserted into pSP-Δ55 (the shuttle plasmid). The DNA sequence of all constructs was confirmed by restriction enzyme digestion, PCR and DNA sequencing (data not shown). Ads were generated by respective homologous recombination between the shuttle plasmid and the packaging plasmid. Identification, purification, and titration (PFU/ml) of the adenovirus were performed as previously described^16^.

### RNA extraction and quantitative reverse transcription PCR (qPCR)

Total RNA was isolated from cells using Trizol (Invitrogen). Two micrograms of RNA was used to synthesize the first single-strand cDNA using RevertAid™ First Strand cDNA Synthesis Kit (Fermentas, Vilnius, Lithuania) as previously reported^43, 44^. Relative quantification of *PRC1* in cells was determined using a Bio-Rad CFX96™ Real-Time PCR Detection System with Power SYBR^®^ Green PCR Master Mix (Applied Biosystems, Carlsbad, CA). *GAPDH* was used as an internal standard. The following primers were used:

For *PRC1*: Forward: 5′-ACA GAC AGA GAC AGA GAT G -3′; Reverse: 5′-GCC GAA TGC TAC TAT TGG -3′

For *GAPDH*: Forward: 5′-GGT GAA GGT CGG AGT CAA CGG A-3′; Reverse: 5′-GAG GGA TCT CGC TCC TGG AAG A-3′

### Immunohistochemical staining

Immunohistochemistry staining was performed and analyzed using Histo-score by two individuals through a double-blind procedure as reported^45, 46^.

### Cell viability assays

The MTT and trypan blue staining assays were performed using standard procedures as we previously reported^16^.

### Cell synchronization

Cells were treated with 150 nM of VX-680 or 2 nM of nocodazole for 16 h (−16 h); the Ads were added to the media at 8 h before the completion of synchronization (−8 h); 8 h later, fresh VX-680/nocodazole-free media with or without taxol/epothilone B/vincristine replaced the media to release the cells from synchronization (0 h). To re-induce mitotic exit, 2 or 3 h after releasing from synchronization (2 h/3 h), the cells were re-treated with VX-680^25^.

### Determination of synergism

Synergism was determined using the software package Calcusyn (Biosoft, Cambridge, UK). A combination index (CI) less than 0.9 was defined as synergism.

### Western blot

Western blots were performed with standard procedures as previously reported^47, 48^, and antibody information is provided in Table S1 of the Supplementary Material.

### In vitro nuclei and microtubules staining

Cells were seeded on glass cover slips, and 24 h later they were infected with Ads at MOI of 1. Cells were then fixed with 4% paraformaldehyde, and co-stained with Phalloidin Alexa Fluor 555 (Molecular Probes, Eugene, OR, USA) and DAPI (Sigma-Aldrich, St. Louis, MO, USA). Cells were observed using a Leica TCS SP2 confocal microscope (Leica Microsystems, Wetzlar, Germany).

### Animal experiments

Animal experiments were performed according to the Guide for the Care and Use of Laboratory Animals set forth by the Huazhong University of Science and Technology. Male BALB/c nude mice (4-week-old) were purchased from the Beijing HFK Bioscience (Beijing, China).

For subcutaneous xenograft model, 2×10^6^ HepG2 or HuH-7 cells were subcutaneously injected into the right flank of each mouse. When tumors reached 80 mm^3^ or 200 mm^3^ in volume, mice were randomly divided (six mice per group). 2×10^9^ PFUs of Ads per mouse or PBS (for control group) were injected into the tumor once every 2 days, for 4 times. Twenty-four hours after the first injection of Ads, some mice received taxol (6 mg/kg body weight intraperitoneal injection, twice a week for 2 weeks). Tumor volume was measured every 3 days and calculated as the length (mm) × width (mm) × depth/2 (mm) as previously reported^49^.

For orthotopic HCC xenograft model, 5×10^5^ HepG2 cells were injected into the right liver lobe in situ, and the mice were subsequently divided (six mice per group) randomly. 2×10^9^ PFUs per day of Ad-vector or Ad-shPRC1 were administrated via tail vein for four times that began at the seventh day after cells implantation. Mice were sacrificed 3 weeks after adenovirus delivery, and blood samples were collected and analyzed for AST and ALB using a BN ProSpec System (Siemens, Germany).

### Statistics

All data are expressed as the mean ± SD and were analyzed using independent sample *t* tests and one-way analyses of variance using SPSS Base 10.0. Results were considered statistically significant when *P* < 0.05.

## Electronic supplementary material

Supplementary Material for Reducing protein regulator of cytokinesis 1 as a prospective therapy for hepatocellular carcinoma

## References

[1] Forner A, Llovet JM, Bruix J (2012). Hepatocellular carcinoma. Lancet.

[2] Kavallaris M (2010). Microtubules and resistance to tubulin-binding agents. Nat. Rev. Cancer.

[3] Liu X, Gong H, Huang K (2013). Oncogenic role of kinesin proteins and targeting kinesin therapy. Cancer Sci..

[4] Gascoigne KE, Taylor SS (2008). Cancer cells display profound intra- and interline variation following prolonged exposure to antimitotic drugs. Cancer Cell.

[5] Orth JD (2008). Quantitative live imaging of cancer and normal cells treated with Kinesin-5 inhibitors indicates significant differences in phenotypic responses and cell fate. Mol. Cancer Ther..

[6] Kops GJ, Weaver BA, Cleveland DW (2005). On the road to cancer: aneuploidy and the mitotic checkpoint. Nat. Rev. Cancer.

[7] Huang HC, Shi J, Orth JD, Mitchison TJ (2009). Evidence that mitotic exit is a better cancer therapeutic target than spindle assembly. Cancer Cell.

[8] Sackton KL (2014). Synergistic blockade of mitotic exit by two chemical inhibitors of the APC/C. Nature.

[9] Fry AM, Yamano H (2006). APC/C-mediated degradation in early mitosis: how to avoid spindle assembly checkpoint inhibition. Cell Cycle.

[10] McGrogan BT, Gilmartin B, Carney DN, McCann A (2008). Taxanes, microtubules and chemoresistant breast cancer. Biochim. Biophys. Acta.

[11] Kanehira M (2007). Oncogenic role of MPHOSPH1, a cancer-testis antigen specific to human bladder cancer. Cancer Res..

[12] Jiang W (1998). PRC1: a human mitotic spindle-associated CDK substrate protein required for cytokinesis. Mol. Cell.

[13] Fu C (2007). Mitotic phosphorylation of PRC1 at Thr470 is required for PRC1 oligomerization and proper central spindle organization. Cell Res..

[14] Shimo A (2007). Elevated expression of protein regulator of cytokinesis 1, involved in the growth of breast cancer cells. Cancer Sci..

[15] Chen J (2016). The microtubule-associated protein PRC1 promotes early recurrence of hepatocellular carcinoma in association with the Wnt/beta-catenin signalling pathway. Gut.

[16] Liu X (2014). MPHOSPH1: a potential therapeutic target for hepatocellular carcinoma. Cancer Res..

[17] Marone M (2002). Survival and cell cycle control in early hematopoiesis: role of bcl-2, and the cyclin dependent kinase inhibitors P27 and P21. Leuk. Lymphoma.

[18] Forys JT (2014). ARF and p53 coordinate tumor suppression of an oncogenic IFN-beta-STAT1-ISG15 signaling axis. Cell Rep..

[19] Li Z (2016). Cdkn2a suppresses metastasis in squamous cell carcinomas induced by the gain-of-function mutantp53(R172H). J. Pathol..

[20] Orth JD, Loewer A, Lahav G, Mitchison TJ (2012). Prolonged mitotic arrest triggers partial activation of apoptosis, resulting in DNA damage and p53 induction. Mol. Biol. Cell.

[21] Hewitt L (2010). Sustained Mps1 activity is required in mitosis to recruit O-Mad2 to the Mad1-C-Mad2 core complex. J. Cell Biol..

[22] Giovinazzi S, Bellapu D, Morozov VM, Ishov AM (2013). Targeting mitotic exit with hyperthermia or APC/C inhibition to increase paclitaxel efficacy. Cell Cycle.

[23] Straight AF (2003). Dissecting temporal and spatial control of cytokinesis with a myosin II Inhibitor. Science.

[24] Perez EA, Patel T, Moreno-Aspitia A (2010). Efficacy of ixabepilone in ER/PR/HER2-negative (triple-negative) breast cancer. Breast Cancer Res. Treat..

[25] Zhu Y, Zhou Y, Shi J (2014). Post-slippage multinucleation renders cytotoxic variation in anti-mitotic drugs that target the microtubules or mitotic spindle. Cell Cycle.

[26] Wang H (2012). CGK733 enhances multinucleated cell formation and cytotoxicity induced by taxol in Chk1-deficient HBV-positive hepatocellular carcinoma cells. Biochem. Biophys. Res. Commun..

[27] Bollrath J (2009). gp130-mediated Stat3 activation in enterocytes regulates cell survival and cell-cycle progression during colitis-associated tumorigenesis. Cancer Cell.

[28] Zhong Z, Wen Z, Darnell JE (1994). Stat3: a STAT family member activated by tyrosine phosphorylation in response to epidermal growth factor and interleukin-6. Science.

[29] Pardanani A (2009). CYT387, a selective JAK1/JAK2 inhibitor: in vitro assessment of kinase selectivity and preclinical studies using cell lines and primary cells from polycythemia vera patients. Leukemia.

[30] Shin DS (2009). Cryptotanshinone inhibits constitutive signal transducer and activator of transcription 3 function through blocking the dimerization in DU145 prostate cancer cells. Cancer Res..

[31] Brosh R, Rotter V (2009). When mutants gain new powers: news from the mutant p53 field. Nat. Rev. Cancer.

[32] Blagosklonny MV (2007). Mitotic arrest and cell fate: why and how mitotic inhibition of transcription drives mutually exclusive events. Cell Cycle.

[33] Derdak Z (2013). Inhibition of p53 attenuates steatosis and liver injury in a mouse model of non-alcoholic fatty liver disease. J. Hepatol..

[34] Bhat KM, Setaluri V (2007). Microtubule-associated proteins as targets in cancer chemotherapy. Clin. Cancer Res..

[35] Rath O, Kozielski F (2012). Kinesins and cancer. Nat. Rev. Cancer.

[36] Chen NC, Chyau CC, Lee YJ, Tseng HC, Chou FP (2016). Promotion of mitotic catastrophe via activation of PTEN by paclitaxel with supplement of mulberry water extract in bladder cancer cells. Sci. Rep..

[37] Dudgeon C (2010). PUMA induction by FoxO3a mediates the anticancer activities of the broad-range kinase inhibitor UCN-01. Mol. Cancer Ther..

[38] Karp JE (2011). Phase 1 and pharmacokinetic study of bolus-infusion flavopiridol followed by cytosine arabinoside and mitoxantrone for acute leukemias. Blood.

[39] Ganem NJ (2014). Cytokinesis failure triggers hippo tumor suppressor pathway activation. Cell.

[40] Rieder CL, Maiato H (2004). Stuck in division or passing through: what happens when cells cannot satisfy the spindle assembly checkpoint. Dev. Cell.

[41] Chen H (2015). Apelin protects against acute renal injury by inhibiting TGF-beta1. Biochim. Biophys. Acta.

[42] Liu XR (2012). A new oncolytic adenoviral vector carrying dual tumour suppressor genes shows potent anti-tumour effect. J. Cell. Mol. Med..

[43] Zhang Y (2017). ANGPTL8 negatively regulates NF-kappaB activation by facilitating selective autophagic degradation of IKKgamma. Nat. Commun..

[44] Liu S (2017). Glyceraldehyde-3-phosphate dehydrogenase promotes liver tumorigenesis by modulating phosphoglycerate dehydrogenase. Hepatology.

[45] Chen H (2014). Apelin inhibits the development of diabetic nephropathy by regulating histone acetylation in Akita mouse. J. Physiol..

[46] Budwit-Novotny DA (1986). Immunohistochemical analyses of estrogen receptor in endometrial adenocarcinoma using a monoclonal antibody. Cancer Res..

[47] Li J (2012). Accumulation of endoplasmic reticulum stress and lipogenesis in the liver through generational effects of high fat diets. J. Hepatol..

[48] Chen H (2017). ELABELA and an ELABELA fragment protect against AKI. J. Am. Soc. Nephrol.: JASN.

[49] Liu X (2012). Gene-viro-therapy targeting liver cancer by a dual-regulated oncolytic adenoviral vector harboring IL-24 and TRAIL. Cancer Gene Ther..

