# Supplementary material for: Reducing protein regulator of cytokinesis 1 as a prospective therapy for hepatocellular carcinoma
Source: Cell Death Dis. 2018 May 10;9(5):534. doi: 10.1038/s41419-018-0555-4 (PMC5945625; doi:10.1038/s41419-018-0555-4)
Supplement: Supplementary file 1 — Supplementary Material for Reducing protein regulator of cytokinesis 1 as a prospective therapy for hepatocellular carcinoma [file 41419_2018_555_MOESM1_ESM.doc]

**Supplementary Material**

*for*

**Reducing protein regulator of cytokinesis 1 as a prospective therapy for hepatocellular carcinoma**

Xinran Liu^1, 2^, Yangkai Li^3^, Lijing Meng^1^, Xin-Yuan Liu^4^, Anlin Peng^5^, Yuchen Chen^1^, Chengyu Liu^1, 2^, Hong Chen^1^, Sheng Sun^1^, Xiaoping Miao^6^, Yu Zhang^1^, Ling Zheng^7^ & Kun Huang^1, 2*^

^1^ Tongji School of Pharmacy, Huazhong University of Science & Technology, Wuhan, China, 430030;

^2^ Centre for Biomedicine Research, Wuhan Institute of Biotechnology, Wuhan, China, 430074;

^3^ Tongji Hospital, Tongji Medical College, Huazhong University of Science & Technology, Wuhan, China, 430030;

^4^ Institute of Biochemistry and Cell Biology, Shanghai Institutes for Biological Sciences, Chinese Academy of Sciences, Shanghai, China, 200031;

^5^ The Third Hospital of Wuhan, Wuhan, China, 430060;

^6^ Tongji School of Public Health, Huazhong University of Science & Technology, Wuhan, China, 430030;

^7^ College of Life Sciences, Wuhan University, Wuhan, China, 430072

^*^**Corresponding author:**

Kun Huang, Ph.D.

13 Hang Kong Road, Wuhan, 430030, P.R. China

Tel/Fax 86-27-83691499

E-mail: [kunhuang@hust.edu.cn](mailto:kunhuang@hust.edu.cn)

The supplementary materials provide 2 tables and 4 figures.

**Supplementary Tables**

**Table S1. List of antibodies used in the present study.**

| Cat. No | Antigen | Company |
| --- | --- | --- |
| ab51248 | PRC1 | Abcam |
| A5316 | β-actin | [Sigma-Aldrich](https://www.baidu.com/link?url=jLBCrnXLR4AqpW3VTXqYh5z2VA04R3qCzsqd5e2WA_CBDACu4pn_DV8fuP3jwxjT&wd=&eqid=fd4343d700013eb50000000359cf07ac) |
| sc-99 | p53 | Santa Cruz Biotechnology |
| sc-752 | cyclin B1 | Santa Cruz Biotechnology |
| sc-271028 | caspase-3 | Santa Cruz Biotechnology |
| 2946 | p21 | Cell Signaling Technology |
| 12640 | STAT3 | Cell Signaling Technology |
| 2407 | p14ARF (p14) | Cell Signaling Technology |
| 4113 | p-STAT3 | Cell Signaling Technology |
| 2926 | cyclin D1 | Cell Signaling Technology |
| 2936  53348 | cyclin D3  p-H3 | Cell Signaling Technology  Cell Signaling Technology |

**Table S2. Features and relative expression levels of PRC1 in clinical samples**

| No. of HCC tissues | Edmondson-Steiner grading | H-score of PRC1 | No. of non-tumor tissues | Original tissues | H-score of PRC1 |
| --- | --- | --- | --- | --- | --- |
| T1 | III | 4 | N1 | tumor adjacent tissue | 1 |
| T2 | III | 1.8 | N2 | tumor adjacent tissue | 1 |
| T3 | III | 6 | N3 | tumor adjacent tissue | 1 |
| T4 | II | 3 | N4 | tumor adjacent tissue | 1 |
| T5 | III | 4 | N5 | tumor adjacent tissue | 5 |
| T6 | III | 4 | N6 | tumor adjacent tissue | 2 |
| T7 | II/III | 3 | N7 | tumor adjacent tissue | 3 |
| T8 | III/IV | 4.2 | N8 | tumor adjacent tissue | 3.2 |
| T9 | II | 0 | N9 | tumor adjacent tissue | 1 |
| T10 | II | 2.4 | N10 | tumor adjacent tissue | 3 |
| T11 | II | 0 | N11 | tumor adjacent tissue | 1 |
| T12 | IV | 3 | N12 | tumor adjacent tissue | 1.4 |
| T13 | III | 3.6 | N13 | tumor adjacent tissue | 1 |
| T14 | II/III | 1 | N14 | tumor adjacent tissue | 3 |
| T15 | I | 2 | N15 | tumor adjacent tissue | 0.3 |
| T16 | II | 3.2 | N16 | tumor adjacent tissue | 1 |
| T17 | III | 5.1 | N17 | tumor adjacent tissue | 3 |
| T18 | III | 3.6 | N18 | tumor adjacent tissue | 0 |
| T19 | IV | 4 | N19 | tumor adjacent tissue | 3.6 |
| T20 | II | 0.3 | N20 | tumor adjacent tissue | 0 |
| T21 | I | 4 | N21 | tumor adjacent tissue | 2 |
| T22 | I | 2.55 | N22 | tumor adjacent tissue | 5 |
| T23 | I | 5 | N23 | tumor adjacent tissue | 3.7 |
| T24 | I | 1.65 | N24 | tumor adjacent tissue | 0 |
| T25 | III | 0.55 | N25 | tumor adjacent tissue | 4.6 |
| T26 | II | 5 | N26 | tumor adjacent tissue | 0.9 |
| T27 | II | 3 | N27 | tumor adjacent tissue | 0.7 |
| T28 | II | 5 | N28 | tumor adjacent tissue | 0 |
| T29 | IV | 4 | N29 | tumor adjacent tissue | 0.1 |
| T30 | III | 4.8 | N30 | tumor adjacent tissue | 3.5 |
| T31 | IV | 4.4 | N31 | tumor adjacent tissue | 0 |
| T32 | II | 4 | N32 | tumor adjacent tissue | 0 |
| T33 | II | 4.2 | N33 | tumor adjacent tissue | 0.3 |
| T34 | II | 0 | N34 | tumor adjacent tissue | 0 |
| T35 | III | 4.3 | N35 | tumor adjacent tissue | 0.1 |
| T36 | IV | 0 | N36 | tumor adjacent tissue | 0 |
| T37 | I | 3 | N37 | cirrhosis | 0.5 |
| T38 | II | 1.8 | N38 | choledocholithiasis | 1 |
| T39 | II | 2.7 | N39 | choledocholithiasis | 0 |
| T40 | III | 3 | N40 | choledocholithiasis | 0 |
| T41 | III | 5 | N41 | choledocholithiasis | 0 |
| T42 | IV | 5 | N42 | cavernous hemangioma | 2.16 |
| T43 | IV | 6 | N43 | cavernous hemangioma | 0 |
| T44 | II/III | 4.5 | N44 | cavernous hemangioma | 4.8 |
| T45 | III | 0.5 | N45 | cavernous hemangioma | 0 |
| T46 | II | 6 | N46 | cavernous hemangioma | 0.8 |
| T47 | IV | 2.5 | N47 | cavernous hemangioma | 6 |
| T48 | IV | 3 | N48 | cavernous hemangioma | 4.5 |
| T49 | IV | 3 | N49 | hepatapostema | 0 |
| T50 | III | 3.5 | N50 | cholangitis | 4.2 |


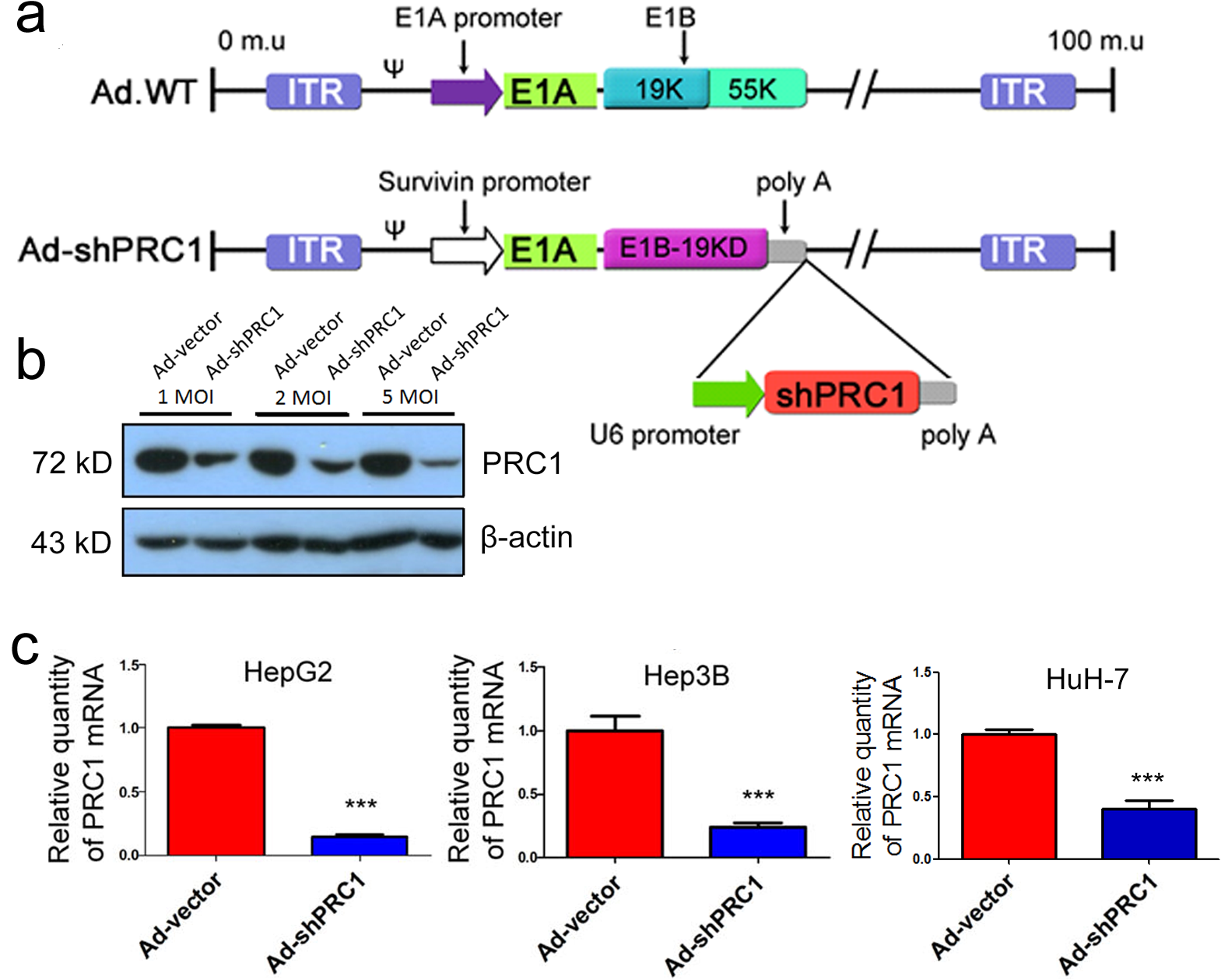


**Figure S1.** Efficiency of Ad-shPRC1 in HCC cells. (**a**) A schematic drawing of the Ad-shPRC1. The promoter of the adenoviral E1A of the Ad-WT was replaced with the cancer cell promoter derived from the *Survivin* gene and the part E1B coding sequence (55K) was also deleted. Ψ, the encapsidation signal; ITR, the inverted terminal repeats. (**b**) Western blot for PRC1 in HepG2 cells 48 hours after infected with Ads of different MOIs. (**c**) Quantification of the *PRC1* transcript level in HepG2, Hep3B and HuH-7 cells 48 hours after infection with Ad-vector and Ad-shPRC1. MOI=1. n = 3 independent experiments.


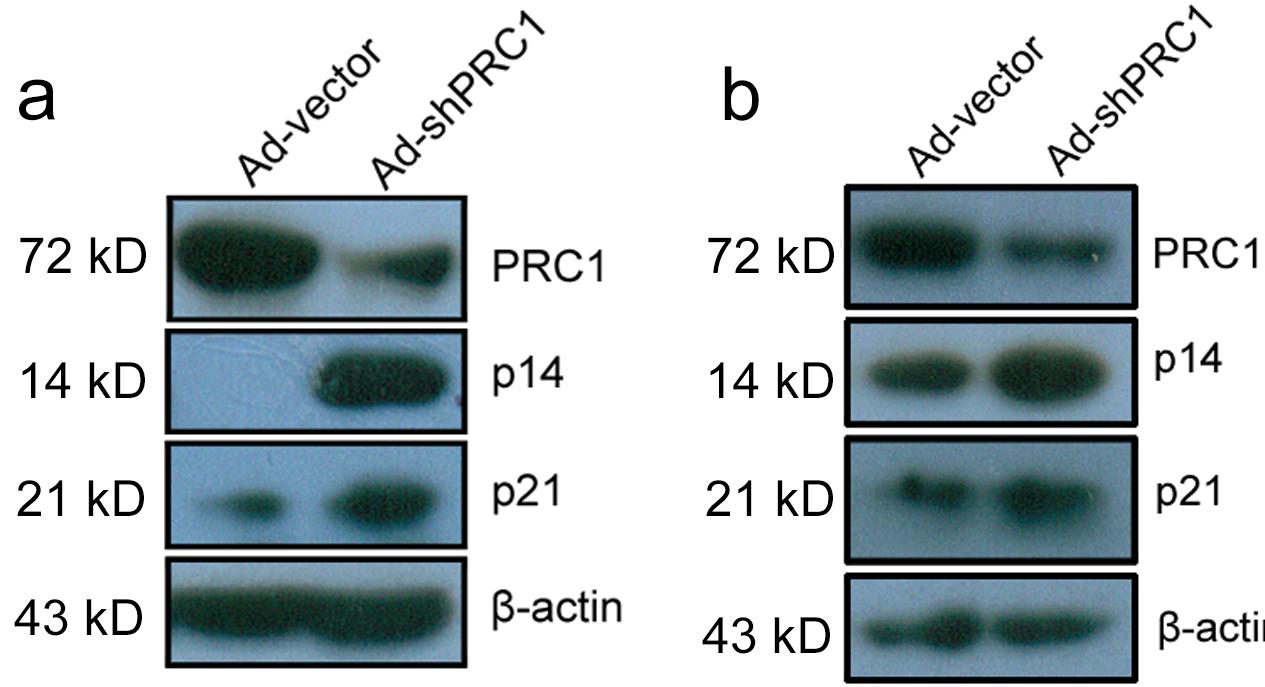


**Figure S2.** PRC1 regulates p53/p14ARF in p53-null and p53-mutant HCC cells. Western blots for indicated proteins receiving Ad-shPRC1 or Ad-vector in Hep3B cells (**a**) and HuH-7 cells (**b**).


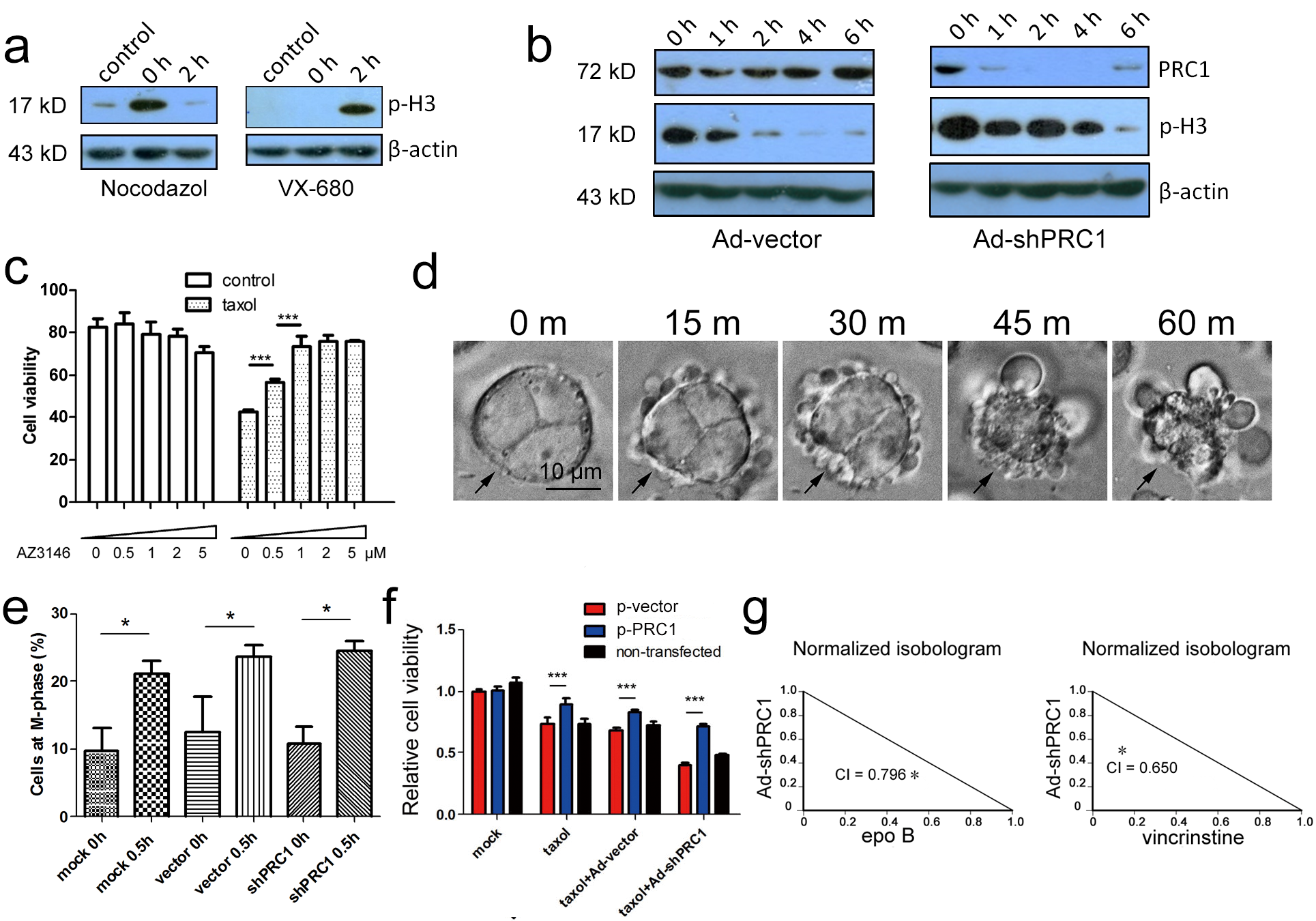


**Figure S3.** Reducing PRC1 blocks mitotic exit at telophase. (**a**) Western blot showing temporal variation of phosphorylated H3 after synchronization by nocodazole (left) or VX-680 (right) in HepG2 cells. (**b**) Western blots for PRC1 at different experiment period after [cell synchronization](http://www.baidu.com/link?url=RVIAdefQ6VkuXVYFLJTPyG3nFBvvQWz7XduC9g68wseRaE8e5U7wA-_fvAvk0NJg8qo8O0iiRAxRF0PSwObcWXvbd79vcJC3iHvZzjVRW38Wl7f_teVBF2Y0h_ZnpQ9U). (**c**) MTT results for HepG2 cells treated with AZ3146 or AZ3146 plus taxol. n = 3 independent experiments. (**d**) Representative pictures of the time lapse phase-contrast microscopy showing the cell cycle progression after infected with Ad-shPRC1. The arrow indicates the multinucleate cell undergoing apoptosis. (**e**) Percentage of cells at M-phase 0.5 hour after G2/M synchronization. Results respond to 3 slides of each group. (**f**) Cell viability of HepG2 cells with indicated treatments using MTT assays. Value of cells transfected with p-vector was arbitrarily set at 1. n = 3 independent experiments. (**g**) The combination index of Ad-shPRC1/epothilone B (epo B) (left) or Ad-shPRC1/vincrinstine (right) on HepG2 cells. (**p* < 0.05, ****p* < 0.001)


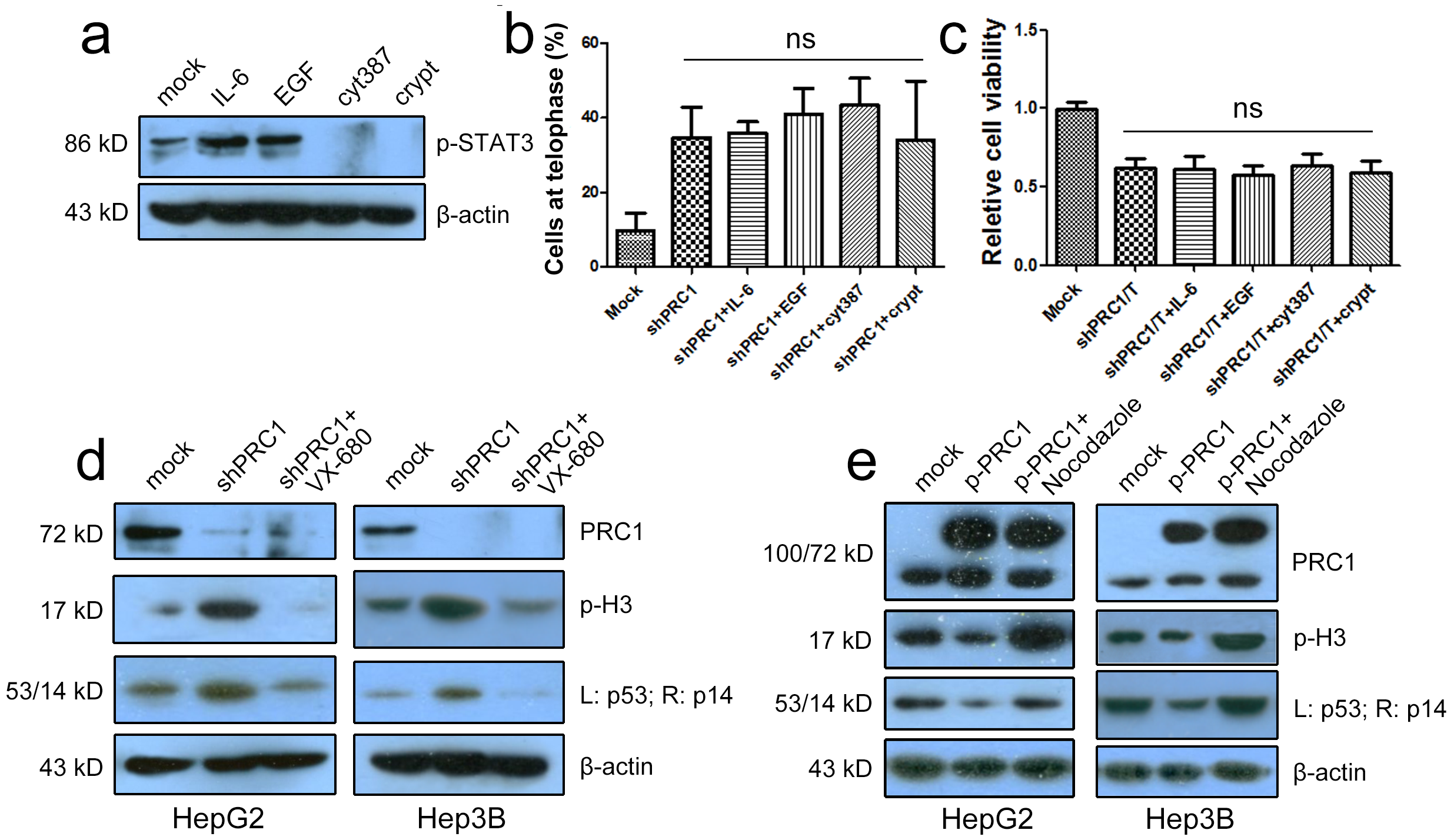


**Figure S4.** STAT3 and p53 are regulated by PRC1 through mitosis regulation. (**a**) Western blots show the level of STAT3 phosphorylation of HepG2 cells by indicated treatments. (**b**) Percentage of cells arrested at telophase under indicated treatments. (**c**) Relative cell viability of HepG2 cells receiving indicated treatments using MTT assays. Value of mock cells was arbitrarily set at 1. n = 3 independent experiments. (**d**) & (**e**) Western blots for indicated proteins in HepG2 and Hep3B cells receiving indicated treatments.
